# Monitoring for Rare Events in a Wireless Powered Communication mmWave Sensor Network

**DOI:** 10.3390/s20123341

**Published:** 2020-06-12

**Authors:** Michael Koutsioumpos, Evangelos Zervas, Efstathios Hadjiefthymiades, Lazaros Merakos

**Affiliations:** 1 Deptament of Informatics and Telecommunications, National and Kapodistrian University of Athens, 157 72 Athens, Greece; mkouts@di.uoa.gr (M.K.); shadj@di.uoa.gr (E.H.); merakos@di.uoa.gr (L.M.); 2 Deptament of Electrical and Electronics Engineering, University of West Attica, 122 44 Egaleo, Greece

**Keywords:** wireless powered communication network (WPCN), energy beamforming, mmWave sensor network

## Abstract

The use of a wireless sensor network to monitor an area of interest for possible hazardous events has become a common practice. The difficulty of replacing or recharging sensor batteries dictates the use of energy harvesting as a means to extend the network’s lifetime. To this end, energy beamforming is used in a millimeter wave wireless power sensor network with randomly deployed nodes. A simple protocol is proposed that allows nodes to report their charging conditions in an effort to select efficient energy-beamforming strategies. Analytical expressions for the probability of successful information reception and successful reporting are provided for two benchmark schemes: the random and the circular energy-beamforming scheme. A Markov chain is used for the former to model the energy level of sensor nodes. Simple sector selection strategies are presented and their performance, in terms of delay and failure information delivery, is assessed through simulations.

## 1. Introduction

The Internet of Things (IoT) aims to connect most electrical devices that people use in their everyday life to the Internet. A combination of mobile applications and cloud infrastructure will allow users to remotely control these devices and automate their functionality based on their needs. Existing applications such as Google Home and Amazon Alexa are the first steps towards the IoT. Wireless sensor networks are an example that will play a key role in the deployment of next-generation mobile networks and the IoT. Since the number of sensors connected directly to a mobile station is expected to grow exponentially in the next few years, the requirements for data rates, latency, and scheduling are essential in the design of those networks. Wireless sensor networks collect information by monitoring the environment through several sensors. Sensors are equipped with the appropriate circuits and antennas to be able to wirelessly transfer their data. For a critical event, the sensor node must respond immediately and provide real-time information on the current situation.

A fundamental limitation of wireless sensors is the use of a battery. Batteries’ limited lifetime and the difficulty of replacing or recharging sensor batteries increase the complexity and operational cost of the sensor network. Recently, energy harvesting (EH) technologies have been proposed [1,2,3] as a solution to extend battery lifetime. Magnetic induction and radio-frequency-based wireless power transfer (WPT) are the main energy-harvesting techniques. The former can be used effectively only on transmission ranges of a few centimeters, and it has been successfully commercialized in some products, including charging pads for mobile phones and medical implants. Radio frequency (RF) WPT, on the other hand, supports relatively long transmission ranges, suitable for IoT devices, such as wireless power sensors, located in a dense area. However, to efficiently charge wirelessly, via RF WPT, a wireless power sensor network, the design of access point (AP) antennas is crucial [4,5,6,7]. Millimeter wave (mmWave) antenna design can provide the required high gains and is suitable for dense network topologies of a few meters. Additionally, the mmWave protocol structure is embedded in the design of next-generation networks providing low-latency, high-bandwidth services. There are two architectures [8,9] for RF WPT: wireless powered communication networks (WPCNs) and simultaneous wireless information and power transfer (SWIPT). In WPCNs, the downlink energy signal is used only to charge user devices. Any information from a user device to the AP is transmitted via the uplink communication link. On the other hand, SWIPT transfers energy and information via the downlink energy signal. Existing works on the use of mmWave technology in WPT focus on the antenna design, channel modeling, and performance evaluation of various SWIPTs schemes. For wireless power sensor networks, the existing works propose various schemes, to increase the distributed estimation performance of the monitored parameter.

Motivated by existing works, we focus on the communication performance aspect of a WPCN network used to monitor an area of interest for possible events. The objective is to deliver the information message (in case of an event) successfully and with the minimum delay possible. Throughout the paper, we consider a mmWave wireless power sensor network with randomly deployed sensor nodes. An AP at the center of the sensing area is responsible for wirelessly charging the sensors and collecting their information. Sensors monitor the environment for possible events that, upon detection, should be reported with minimum delay. We introduce an optional reporting phase during which sensors, with energy storage exceeding a predefined threshold, report their status to the AP. Partial knowledge of the sensors energy status may prove helpful for the AP to select alternative and more efficient energy-beamforming strategies. Moreover, since transmissions in the mmWave band are susceptible to blockage, we consider a flooding technique for information message delivery. That is, nodes that intercept the message become information transmitters themselves in an effort to increase the reliability of the system and minimize the delivery delay.

Specifically, the main contributions of this paper are as follows:

- We model the energy level of a sensor battery as a finite Markov chain and derive its steady-state distribution for various blocking probabilities under the assumption of random energy beamforming;
- In case of an event, we derive an analytical expression for the probability of successful information reception, using stochastic geometry tools for two cases: random and circular selection of sectors;
- We provide the probability that the sensors’ status report messages are received correctly by the AP for the two cases mentioned above;
- Simple sector selection strategies are presented and their performance is assessed through simulations. Taking into account the number of reports received by the nodes of each sector, two strategies are proposed that identify and subsequently degrade energy beamforming towards sectors with heavy blockage. Periodic shifting of sectors is another proposed scheme that aims to boost nodes located at the edges of the beamforming pattern.

The rest of this paper is organized as follows: Section 2 summarizes the existing research work. In Section 3, we present the system model and provide analytical expressions for the probability of successful information reception and successful reporting based on the proposed reporting scheme. Alternative energy-beamforming strategies that utilize node reporting are presented in Section 4. Numerical and simulation results are presented in Section 5. Finally, Section 6 contains our conclusions.

## 2. Prior Work

There are several recent publications in wireless power transfer in the millimeter wave band. The work in [4] focused on the performance of WPT in mmWave massive multiple-input–multiple-output (MIMO) systems. Results showed that the harvested energy at each piece of user equipment (UE) increases linearly with the number of antennas at the access point. Moreover, in [5], the authors studied the required number of RF chains to achieve a fully digital beamforming performance, based on a hybrid analog/digital beamforming with a limited number of RF chains. The work in [6] considered a SWIPT scheme in massive MIMO–NOMA systems, to achieve the optimal trade-off between spectrum and energy efficiency. The work in [7] studied the performance of SWIPT with 5G frequencies at 3.5 and 28 GHz and proposed an architecture that increases the throughput of cell-edge users and improves the energy-harvesting efficiency of cell-center users. On the performance of SWIPT schemes for mm-wave communication systems, the authors of [10] considered two channel models to accurately model the signal propagation process in mm-wave bands, namely, two-wave with diffused power and fluctuating two-ray.

Sensor positioning information is crucial to the performance of sensor networks, especially when the sensors are deployed in harsh environments. The authors of [11] presented recent advances in the field of wireless positioning under sensor mobility, highlighting the importance of mobility exploitation, sensor cooperation in terms of processing power, and advanced signal processing techniques. Reliable positioning in a multipoint connectivity scenario was considered in [12]. As it was shown, the use of coordinated multipoint reception increases positioning accuracy considerably. The target tracking problem was considered in [13]. The authors proposed a novel mmWave beamforming antenna system capable of tracking targets in a wide area, and its performance was tested in an indoor scenario.

Sensing area partitioning and energy coverage are important factors for the reliability and efficiency of the network.The authors of [14] investigated the energy coverage of a mmWave small-cell network by modeling the network as a homogeneous Poisson point process (HPPP) and applying stochastic geometry. An energy-efficient coverage algorithm was presented in [15], aiming for a balance between coverage rate and energy cost by adjusting the sensing radius of each sensor node. On the other hand, aiming for energy economy of the sensors, a well-organized sensor network is required to implement energy-efficient routing protocols. Clustering methods can easily organize the network and reduce energy consumption by controlling the intracluster communications. The authors of [16,17] considered methods to optimally select the cluster sensor head.

The work in [18] investigated directional WPT for a large-scale network consisting of multiple mmWave APs and devices. The authors proposed a directional power transfer scheme where the AP charges the devices falling in the beamforming direction in which they receive the maximum power. The work in [19] focused on the performance of wireless powered sensor networks. Assuming the sensors are spatially distributed in the cell around the AP, the authors presented analytical expressions of the energy outage probability of a sensor as well as the beam outage probability. In the same paper, several location-aware sector selection schemes were presented where the AP selects a sector based on the number of sensors in that area. The work in [20] considered a system that consists of multiple sensors that are deployed to obtain information about a common parameter of interest. They examined the beam pattern selection and charging power allocation with considerations on the distributed estimation performance of the monitor parameter. Finally, in [21], the authors studied the security of communications in downlink SWIPT mmWave ultra-dense networks, where secrecy performance is derived under a stochastic geometry framework.

In [22], the authors presented the use of unmanned aerial vehicles (UAV) in wireless communications. They proposed a hierarchical architecture with multilayer and distributed features for next-generation wireless communication networks and power transfer.

The work presented in this paper was inspired by [19]. However, the problem considered in [19] is the periodic transmission of information by sensor nodes which, after charging for a constant time period, transmit using all their available stored energy. Moreover, only random energy beamforming is considered in [19]. By contrast, we consider a WPCN that monitors an area for rare events. Sensor nodes do not need to transmit unless they have detected an event or they have overheard an information transmission message by other nodes. Ideally, the AP should follow a strategy that will keep as many sensor nodes sufficiently charged, in an effort to increase the probability of successful transmission of the information message.

## 3. Problem Statement and System Model

**Figure 1:**
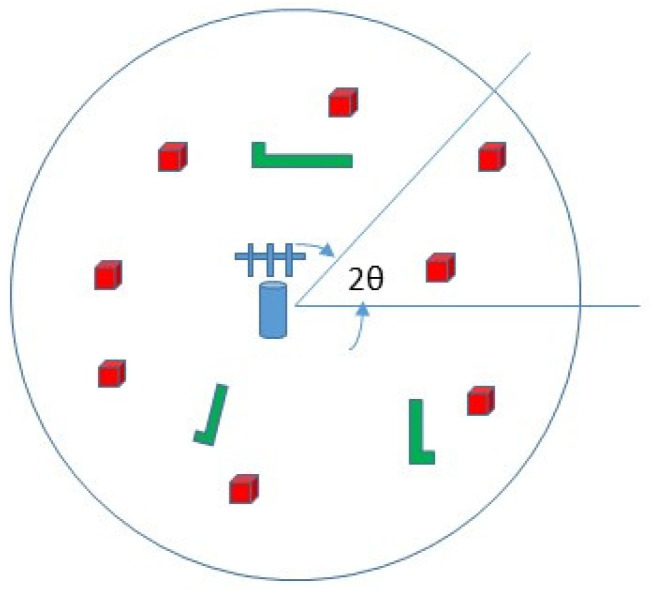
Network topology. Nodes are depicted with boxes (red) and obstacles are depicted with L-shaped schemes (green).

We envisage a mmWave WPCN with randomly deployed sensor nodes in a single cell, modeled by a disc of radius ρ. An AP, centered at the origin of the cell, is used to wirelessly charge the nodes and to collect information by them. The nodes sense the environment for possible events, which upon detection should be reported to the AP with the minimum delay possible, e.g., hazardous phenomena such as fire or toxic gas leakage. The AP is equipped with a uniform linear array of *M* antennas and switches the radiation beam among *S* predefined sectors, which cover the whole cell. Beamforming, for energy transfer and information retrieval, is performed every $T_{s}$ seconds. The topology of the system is shown schematically in Figure 1.

To model the blockage effect, we assume that the line-of-sight probability decays exponentially as the distance between the BS and the node increases [23]. That is, the probability of LOS of a node located at a distance $d_{i}$ from the BS is

$$P_{LOS}^{i}=e^{-\beta d_{i}}$$

with the parameter β depending on the density and the geometry of the blocks.

**Figure 2:**
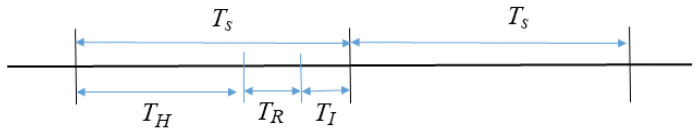
Time slot format.

Time is slotted, and each slot (of duration $T_{s}$) consists of three fields, the harvesting period $T_{H}$, the reporting period $T_{R}$, and the information period $T_{I}$, as is shown in Figure 2.

During the energy-harvesting period, sensors located in the served sector harvest energy and charge their battery. If the stored energy level of a sensor exceeds a threshold, $E_{\tau }$, the sensor reports its condition to the AP using the surplus energy. Reporting takes place during $T_{R}$ and gives a chance to the AP to acquire knowledge for the sensors’ condition and select alternative beamforming strategies. Two benchmark strategies for energy transfer to nodes are considered in this work: random selection and circular selection of sectors to be energy-beamformed. In both cases, the reporting field may be omitted, leaving more time for energy harvesting and/or information transmission in case of an event. We should mention that reporting does not deplete the stored energy, and nodes that have exceeded the energy threshold $E_{\tau }$ reserve this amount of energy for possible information transmission. Upon detection of an event, the node will transmit its ID and proper information regarding the event in the time period $T_{I}$. To increase the probability of correct reception by the AP, the sensor node uses all its available power for information transmission. As reporting is unnecessary in this case, a node holding information continues to charge during the interval $T_{R}$ (instead of transmitting a report) in an effort to maximize its transmission power and, thus, the probability of successful reception of its message.

The information message may not reach the AP for several reasons, such as bad channel condition, blockage of the node, etc. To overcome this problem, we may allow nodes to act as relays. Nodes in the neighbor of the information transmission node intercept the message and retransmit it when their home sector is selected by the AP. This process is repeated and the information message is propagated in an epidemic fashion towards the AP. Although the epidemic dissemination of the information message is not the focus of this work, some simulation results are provided for comparison reasons. To avoid the collision of multiple transmission at the AP, we adopt an orthogonal multiple access scheme, for example, FDMA. The abundance of bandwidth in the mmWave band and the low rate information transmission justify such a choice.

## 3.1. Markov Chain Modeling of Battery Energy Levels

The wirelessly harvested energy by the $k^{th}$-node depends on the received RF power and the time duration the node is exposed to radiation. In the sequel, we adopt the nonlinear model presented in [24], according to which

(1) $$E=\frac{tL(1-\exp\left(-\zeta_{1}P^{RF}\right))}{1+\exp(-\zeta_{1}\left(P^{RF}-\zeta_{2}\right)}$$

where $P^{RF}$ is the received power, *L* is the maximum possible harvested energy, and $\zeta_{1}$, $\zeta_{2}$ are constants related to the circuit implementation. *L* is taken equal to $\gamma E_{b}$, with $E_{b}$ the maximum energy size of the battery and γ∈[0,1] a parameter to model the efficiency of the charging process. In this section, we model the energy level of a node battery as a finite Markov chain (MC). Each battery is of size $E_{b}$ which we discretize in *N* levels, $\epsilon_{i}=iE_{b}/N$, i=0,…,N. The discretization step $E_{s}=E_{b}/N$ is taken equal to the energy needed by the sensor node to sense the environment and to complete necessary processing tasks during a time slot. The node operation depends on whether the node is located in the served sector or not.

- If the node is outside the served sector, then it senses the environment for a period of $T_{H}+T_{R}$, and then it enters the reception mode (for a period $T_{I}$) to listen possible information transmissions by neighboring nodes. To accomplish these tasks, the sensor node depletes one energy unit, that is, $E_{s}=E_{b}/N$;
- If the sensor is located in the sector that the AP is currently pointed to, it harvests energy for a period $T_{H}$, and if its battery level $E_{m}=mE_{s}$ exceeds a threshold $E_{\tau }=\tau E_{s}$, it uses $\left(m-\tau \right)E_{s}$ energy units to report its condition to the AP during the period $T_{R}$. Then, it enters the reception mode for a period $T_{I}$ in order to listen possible information transmissions by neighboring nodes. Sensing and listening costs one unit of energy;
- If the sensor node has detected an event in its sensing area, it awaits until the AP steers its beam towards its host sector. Then, it harvests energy for a period $T_{H}+T_{R}$ and transmits the relative information, during the period $T_{I}$, using all its available energy. In the epidemic information dissemination scenario, neighboring nodes that intercept such a message act as relays and retransmit the message in a following time slot in an effort to increase redundancy and, thus, the reliability of the system. These nodes deplete all the stored energy and start charging again from zero. The detection of an event signals the end of the analysis horizon, and therefore, it is not included in the modeling process.

From the discussion above, it is clear that by the end of each time slot, the sensor battery energy level assumes one of τ+1 possible values, 0,…,τ, with τ<N. Therefore, to model the stored energy process of a sensor node, we use an MC with states $S_{i}$, i=0,…,τ, where each transition takes place at the end of each time slot. The probability that a node lies on the served sector or not depends on the beamforming strategy used by the AP. If the AP selects sectors randomly, then

$$q=P\left\{\mathrm{sector}\;\mathrm{served}\;\mathrm{at}\;\mathrm{time}\;n\right\}=\frac{\theta }{\pi }$$

with 2θ the central angle of a sector. If the AP selects the sectors in a circular fashion, then for a typical node

q(n)=P{sector served at time n}=I(mod(n,S)=0)

where I(·) denotes the indicator function. This choice leads to a nonhomogeneous MC, thus complicating the analysis. To this end, we use basic principles to derive expressions for the involved probabilities of successful information reception and reporting. Assuming that the CDF of the charging process is known, $F_{E}\left(\epsilon \right)=P\left\{E\leq \epsilon \right\}$ the possible transitions between states $S_{i}$, i=0,…,τ of the MC are as follows:

- Transitions $S_{i}\to S_{i-1}$
  $$p_{i,i-1}=1-q+qP\left\{E<E_{s}\right\}=1-q+qF_{E}\left(E_{s}\right)$$
  Note that one unit of energy is consumed for sensing and listening, and therefore, if the sensor node is outside the served sector (with probability 1−q) or the sensor node is inside the serving sector (with probability *q*) and the harvested energy is below $E_{s}$, the node will transit to a lower state;
- Transitions $S_{0}\to S_{0}$
  $$p_{0,0}=1-q+qP\left\{E<2E_{s}\right\}=1-q+qF_{E}\left(2E_{s}\right)$$
- Transitions $S_{i}\to S_{i+j}$
  $$\begin{array}{ccc} p_{i,i+j} & = & qP\{\left(j+1\right)E_{s}\leq E<\left(j+2\right)E_{s}\}\\ & = & q(F_{E}\left(\left(j+2\right)E_{s}\right)-F_{E}\left(\left(j+1\right)E_{s}\right)),\;j=0,\ldots ,\tau -i-1 \end{array}$$
  A transition of this type is possible whenever the sensor node is inside the served sector (with probability *q*) and the harvested energy is $\left(j+1\right)E_{s}$ energy units;
- Transitions $S_{i}\to S_{\tau }$
  $$p_{i,\tau }=qP\left\{E\geq \left(\tau +1-i\right)E_{s}\right\}=q\left(1-F_{E}\left(\left(\tau +1-i\right)E_{s}\right)\right)$$

Having in our disposal the transition probability matrix $P=\{p_{i,j}\}$, we find the steady state distribution ψ of the Markov chain. Using the method presented in [25]

(2) $$\psi ={\left(P^{T}-I+B\right)}^{-1}e$$

with $e={\left[1\ldots 1\right]}^{T}$ and $B_{i,j}=1$, ∀i,j.

To complete the analysis presented in this subsection, we derive the CDF $F_{E}\left(\epsilon \right)$ of the harvested energy. The harvested energy is an increasing function of the received power, $E=h(P^{RF})$, and therefore:

$$P\left\{E\leq \epsilon \right\}=P\{P^{RF}\leq h^{-1}\left(\epsilon \right)\}$$

Solving Equation (1) for $P^{RF}$, we obtain

$$P^{RF}=-\frac{1}{\zeta_{1}}\ln\left(\frac{tL-\epsilon }{tL+\epsilon \exp(\zeta_{1}\zeta_{2})}\right)=\eta \left(t,\epsilon \right)$$

Thus,

$$E\leq \epsilon \Leftrightarrow P^{RF}\leq \eta \left(t,\epsilon \right)$$

For the received power $P^{RF}$, we use the model in [26]. For a node located at distance *r* from the AP and at a normalized angle ϕ from the direction of the serving beam, the received power is

(3) $$P^{RF}=\frac{P_{0}{\left|g\right|}^{2}F_{M}\left(\phi \right)}{1+r^{a}}$$

where $P_{0}$ is the BS transmit power, α is the path loss exponent, and *g* is the complex channel gain between the sensor node and the AP, modeled as complex Gaussian with zero mean and unit variance, i.e., g∼CN(0,1). The function $F_{M}\left(\phi \right)$ represents the Fejér kernel of order *M*, that is

$$F_{M}\left(\phi \right)=\frac{1}{M}\frac{\sin^{2}\left(\frac{\pi M\phi }{2}\right)}{\sin^{2}\left(\frac{\pi \phi }{2}\right)}$$

Therefore, for a harvesting period $T_{H}$, we obtain

(4) $$\begin{array}{ccc} F_{E}\left(\epsilon \right)=P\left\{E\leq \epsilon \right\} & = & P\left\{P^{RF}\leq \eta \left(T_{H},\epsilon \right)\right\}=P\left\{{\left|g\right|}^{2}\leq \frac{\eta \left(T_{H},\epsilon \right)\left(1+r^{a}\right)}{P_{0}F_{M}\left(\phi \right)}\right\}\\ & = & 1-E_{\Phi }\left[\exp\left(-\eta \left(T_{H},\epsilon \right)\frac{1+r^{a}}{P_{0}F_{M}\left(\phi \right)}\right)\right]\\ & = & 1-\frac{1}{\theta \rho^{2}}\int_{-\theta }^{\theta }\int_{0}^{\rho }\exp\left(-\eta \left(T_{H},\epsilon \right)\frac{\left(1+r^{a}\right)}{P_{0}F_{M}\left(\phi \right)}-\beta r\right)rdrd\phi \end{array}$$

The equality in the second line of equations is due to the fact that ${\left|g\right|}^{2}$ is exponentially distributed, whereas the last equation follows by considering the spatial distribution of distances and angles in the serving sector, where energy harvesting takes place. Moreover, in the last equation, we have taken into account the LOS probability of a link.

## 3.2. Information Phase

In this section, we derive analytical expressions for the probability of successful information reception. Two cases are considered: random and circular energy beamforming.

### 3.2.1. Probability of Successful Information Reception in Case of Random Energy Beamforming

When a sensor node detects an event, it must transmit relative information to the AP with the minimum delay possible. When the sensor is in the serving sector, it first charges for a period $T_{H}+T_{R}$ and then transmits, using all its available power, at the end of the time slot over a period $T_{I}$. When the node is outside the serving area, it awaits until the AP switches the beam towards its direction. In the sequel, we find an expression for the probability of successful message delivery. A node holding a message will use all its reserved battery energy to transmit it. Therefore, if the node’s battery is at energy level $mE_{s}$, the transmission power is

$$P_{m}=\frac{mE_{s}}{T_{I}}$$

The signal-to-noise ratio (SNR) at the AP from the $k^{th}$ transmitting sensor is

(5) $${\mathrm{SNR}}_{k}=\frac{P_{k}{\left|g_{k}\right|}^{2}F_{M}\left(\phi_{k}\right)}{\left(1+d_{k}^{a}\right)\sigma^{2}}$$

where $\sigma^{2}$ is the noise power. Therefore,

(6) $$\begin{array}{ccc} P\{\mathrm{SNR}\leq x\;|\;P_{m}\} & = & P\left\{\frac{P_{m}{\left|g\right|}^{2}F_{M}\left(\phi \right)}{\left(1+r^{a}\right)\sigma^{2}}\leq x\;|\;P_{m}\right\}\\ & = & P\left\{{\left|g\right|}^{2}\leq \frac{x\left(1+r^{a}\right)\sigma^{2}}{P_{m}F_{M}\left(\phi \right)}\;|\;P_{m}\right\}\\ & = & 1-E_{\Phi }\left[\exp\left(-x\frac{\left(1+r^{a}\right)\sigma^{2}}{P_{m}F_{M}\left(\phi \right)}\right)\right]\\ & = & 1-\frac{1}{\theta \rho^{2}}\int_{-\theta }^{\theta }\int_{0}^{\rho }\exp\left(-x\frac{\left(1+r^{a}\right)\sigma^{2}}{P_{m}F_{M}\left(\phi \right)}\right)rdrd\phi \end{array}$$

The probability that a node transmits with power $P_{m}$ is

$$p_{m}=P\left\{P_{m}\right\}=\left\{\begin{array}{cc} \sum_{j=0}^{\min\{\tau ,m\}}\psi_{j}(F_{E}\left(\left(m+1-j\right)E_{s}\right)-F_{E}\left(\left(m-j\right)E_{s}\right),\\ \; & \;m=0,\ldots ,N-1\\ \sum_{j=0}^{\tau }\psi_{j}(1-F_{E}\left(\left(N+1-j\right)E_{s}\right),\; & \;m=N \end{array}\right.$$

Note that the CDF $F_{E}\left(\epsilon \right)$ used in the previous formula is given by Equation (4) with $T_{H}+T_{R}$ replacing $T_{H}$. Unconditioning on $P_{m}$, we obtain

(7) $$\begin{array}{c} F_{SNR}\left(x\right)=P\left\{SNR\leq x\right\}=\\ 1-\frac{1}{\theta \rho^{2}}\sum_{m=0}^{N}p_{m}\int_{-\theta }^{\theta }\int_{0}^{\rho }\exp\left(-x\frac{\left(1+r^{a}\right)\sigma^{2}}{P_{m}F_{M}\left(\phi \right)}\right)rdrd\phi \end{array}$$

Successful reception of the information message is possible if

(8) $$R_{I}<W^{\prime }\log_{2}\left(1+\mathrm{SNR}\right)$$

where $R_{I}$ is the information rate and $W^{\prime }$ is the bandwidth allocated to a node. Using orthogonal signaling, we assume that the available bandwidth *W* is equally divided to all nodes, and therefore

$$W^{\prime }=\frac{W}{S_{u}}$$

with $S_{u}$ an upper bound on the total number of nodes. Solving Equation (8) for SNR and using Equation (7), we obtain

(9) $$\begin{array}{c} p_{si}^{r}=P\left\{\mathrm{successful}\;\mathrm{information}\;\mathrm{reception},\;\mathrm{random}\;\mathrm{energy}\;\mathrm{beamforming}\right\}=\\ \frac{1}{\theta \rho^{2}}\sum_{m=0}^{N}p_{m}\int_{-\theta }^{\theta }\int_{0}^{\rho }\exp\left(-\left(2^{R_{I}S_{u}/W}-1\right)\frac{\left(1+r^{a}\right)\sigma^{2}}{P_{m}F_{M}\left(\phi \right)}\right)rdrd\phi \end{array}$$

The information rate $R_{I}$ equals $D_{I}/T_{I}$, where $D_{I}$ is the amount of data (bits) that the node sends to the AP, and $T_{I}$ is the transmission time. In our case, $T_{I}=1-T_{H}-T_{R}$, and therefore, there is a trade-off between the charging time (transmission power) and the information rate.

### 3.2.2. Probability of Successful Information Reception in Case of Circular Energy Beamforming

When the AP beamforms in a round robin fashion and for a static channel, we have to consider two complementary cases. Assuming for simplicity that τ>S, the first case refers to nodes that in one time slot harvest energy greater than $SE_{s}$ energy units. These nodes manage to increase their battery energy level cycle-by-cycle until the threshold $E_{\tau }$ (used for reporting) has been reached. After that, the nodes spend the amount of energy exceeding $E_{\tau }$ for reporting and return to level $E_{\tau }-\left(S-1\right)E_{s}$ before they start to charge again. Note that $\left(S-1\right)E_{s}$ energy units are lost due to the circular beamforming pattern.

The second case refers to nodes that in one time slot harvest energy less than $SE_{s}$ energy units. Due to the circular nature of energy beamforming, these nodes deplete one energy unit per time slot and eventually return to the zero state before they start charging again.

For the probability of successful information reception using the circular energy-beamforming scheme, $p_{si}^{c}$, Equation (9) is still valid, but the transmission powers $P_{m}$ and their corresponding probability $p_{m}=P\left\{P_{m}\right\}$ should be changed. The different cases are summarized in the following array

(10) $$P_{m}=\left\{\begin{array}{ccc} \frac{mE_{s}}{T_{I}}, & p_{m}=F_{E}\left(\left(m+1\right)E_{s}\right)-F_{E}\left(mE_{s}\right), & m=0,\ldots ,S-1\\ \frac{\left(\tau -S+m\right)E_{s}}{T_{I}}, & p_{m}=F_{E}\left(\left(m+1\right)E_{s}\right)-F_{E}\left(mE_{s}\right), & m=S,\ldots ,N+S-1-\tau \\ \frac{NE_{s}}{T_{I}}, & p_{m}=1-F_{E}\left(\left(N+S-\tau \right)E_{s}\right), & m=N+S-\tau ,\ldots ,N \end{array}\right.$$

Note that if no reporting is required, the parameter τ is set equal to *N*. In this case, the information transmission power is $NE_{s}/T_{I}$ with probability $1-F_{E}\left(SE_{s}\right)$. That is, if a sensor node charges in one time slot at energy levels greater than $SE_{s}$, it will transmit the information message in full power.

### 3.2.3. Delay Analysis

In case of an event detection, the detecting node should report to the AP as soon as possible. An information message may reach the AP directly if the transmission of the detecting node is successful, or indirectly by relaying the message through neighboring nodes overhearing the transmission if the epidemic mode is on. This epidemic information dissemination is very robust but complicates the delay analysis. To this end, we present some basic delay analysis results, considering only the case of one-hop transmissions.

#### Delay of First Transmission Attempt for Random Energy Beamforming 

Suppose an event takes place at a random time instance, and a nearby node detects the event. This node will transmit the information message at the end of the current time slot if the node is located in the serving sector, or at the end of an upcoming time slot for which the tagged sector is selected. Since the AP selects randomly the sectors with probability q=θ/π, the time (in slots) until the first visit to the tagged sector is geometrically distributed with success probability *q*. Therefore, the average delay until transmission of the message is

(11) $$\begin{array}{c} E[\mathrm{Delay}\;\mathrm{for}\;\mathrm{random}\;\mathrm{energy}\;\mathrm{beamforming}]=\\ \frac{1}{2}q+\left(1-q\right)\sum_{n=1}^{\infty }\left(\frac{1}{2}+n\right)q{\left(1-q\right)}^{n-1}=\frac{1}{q}-\frac{1}{2}=S-\frac{1}{2} \end{array}$$

where the term 1/2 in the left side of the equation represents the average residual delay, that is, the time elapsed between detection and the end of the current time slot. Note that the first transmission attempt is successful with probability $p_{si}^{r}$.

#### Delay of First Transmission Attempt for Circular Energy Beamforming 

Suppose again that an event takes place at a random time instance and that sectors are selected in a round robin fashion. Then, the average delay is 1/2, which accounts for the average residual delay until the end of the current time slot, plus the average delay of a uniformly distributed random variable over the range [0,…,S−1]. Therefore,

(12) $$E\left[\mathrm{Delay}\;\mathrm{for}\;\mathrm{circular}\;\mathrm{energy}\;\mathrm{beamformin}\right]=\frac{1}{2}+\frac{S-1}{2}=\frac{S}{2}$$

which is almost half the value in Equation (11). Note that in this case, the first transmission attempt is successful with probability $p_{si}^{c}$.

### 3.3. Reporting Phase

The reporting phase is crucial to design alternative charging strategies. Knowledge (or estimation) of the energy reserves of nodes located in a sector may dictate specific actions to be taken by the AP. For example, lack of reports from nodes in the same sector over several time slots is an indicator of heavy blockage. In this case, insisting on beamforming in the specific direction leads to a waste of resources and degrades the overall performance of the system. Nodes in the serving sector, after harvesting energy for a period $T_{H}$, report their ID to the AP provided that their battery energy exceeds a specific threshold $E_{\tau }$. To be more specific, the nodes use the surplus (if any) battery energy $\left(m-\tau \right)E_{s}$, m=τ+1,…,N to report their presence to the AP. Needless to say that the reports are not always delivered to the AP due to the possible low transmitting power, bad channel conditions or link blockage. In the sequel, we estimate the probability of successfully delivering a report, under the assumption that the serving sector is selected randomly and in a circular fashion.

#### 3.3.1. Probability of Successful Reporting in Case of Random Energy Beamforming

It is clear that only nodes with battery energy levels greater that $E_{\tau }=\tau E_{s}$ attempt to transmit report messages. Thus, the probability of reporting is

(13) $$p_{r}^{r}=\sum_{i=0}^{\tau }\psi_{i}P\left\{E>\left(\tau +1-i\right)E_{s}\right\}=\sum_{i=0}^{\tau }\psi_{i}(1-F_{E}\left(\left(\tau +1-i\right)E_{s}\right)$$

Given that a node reports to the AP, the probability that the sent message is received correctly by the AP, $p_{sr}^{r}$, is

(14) $$\begin{array}{ccc} p_{sr}^{r} & = & \frac{1}{\theta \rho^{2}p_{r}^{r}}\sum_{i=0}^{\tau }\psi_{i}\sum_{\ell =1}^{N-\tau }p_{\ell }\\ & & \int_{-\theta }^{\theta }\int_{0}^{\rho }\exp\left(-\frac{1+r^{a}}{F_{M}\left(\phi \right)}\left(\frac{\left(2^{D_{R}/T_{R}W^{\prime }}-1\right)\sigma^{2}}{P_{\ell }}+\frac{\eta_{\tau +1-i}}{P_{0}}\right)-\beta r\right)rdrd\phi \end{array}$$

with

(15) $$p_{\ell }=\left\{\begin{array}{cc} \frac{F_{E}\left(\left(\tau +\ell +1-i\right)E_{s}\right)-F_{E}\left(\left(\tau +\ell -i\right)E_{s}\right)}{1-F_{E}\left(\tau +1-i\right)E_{s}}\; & \ell <N-\tau \\ \frac{1-F_{E}\left(\left(\tau +\ell +1-i\right)E_{s}\right)}{1-F_{E}\left(\left(\tau +1-i\right)E_{s}\right)} & \ell =N-\tau \end{array}\right.$$

The proof is provided in Appendix A.

#### 3.3.2. Probability of Successful Reporting in Case of Circular Energy Beamforming

If nodes are charged in a circular fashion, that is, every *S* time slots, then the probability of reporting is $1-F_{E}\left(\left(S+1\right)E_{s}\right)$ provided that τ≥S and $1-F_{E}\left(\left(\tau +1\right)E_{s}\right)$ otherwise. These two cases are written in compact form as

(16) $$p_{r}^{c}=P\left\{E>\left(\min\left\{S,\tau \right\}+1\right)E_{s}\right\}=1-F_{E}\left(\left(\min\left\{S,\tau \right\}+1\right)E_{s}\right)$$

The reporting nodes form a point process, which is the original PPP thinned by the probability $p_{r}^{c}$. Following the same steps as in Appendix A, the probability that the sent message is received correctly by the AP, $p_{sr}^{c}$, is

(17) $$\begin{array}{ccc} p_{sr}^{c} & = & \frac{1}{\theta \rho^{2}p_{r}^{c}}\sum_{\ell =1}^{N-\tau }p_{\ell }\\ & & \int_{-\theta }^{\theta }\int_{0}^{\rho }\exp\left(-\frac{1+r^{a}}{F_{M}\left(\phi \right)}\left(\frac{\left(2^{D_{R}/T_{R}W^{\prime }}-1\right)\sigma^{2}}{P_{\ell }}+\frac{\eta_{\min\{S,\tau \}+1}}{P_{0}}\right)-\beta r\right)rdrd\phi \end{array}$$

with

(18) $$p_{\ell }=\left\{\begin{array}{cc} \frac{F_{E}\left(\left(\min\left\{S,\tau \right\}+\ell +1\right)E_{s}\right)-F_{E}\left(\left(\min\left\{S,\tau \right\}+\ell \right)E_{s}\right)}{1-F_{E}\left(\min\left\{S,\tau \right\}+1\right)E_{s}}\; & \ell <N-\tau \\ \frac{1-F_{E}\left(\left(\min\left\{S,\tau \right\}+\ell +1\right)E_{s}\right)}{1-F_{E}\left(\left(\min\left\{S,\tau \right\}+1\right)E_{s}\right)} & \ell =N-\tau \end{array}\right.$$

## 4. Sector Selection Strategies

In this section, we describe strategies that the AP can follow to select a sector for charging. It is clear that these strategies utilize the feedback provided by the reporting messages. The reporting or “presence” messages designate the number of nodes that have reached the charging energy threshold $E_{\tau }$. A large number of successfully received reports from nodes in a sector indicates that the sector is sufficiently covered by charged nodes. Therefore, it is expected that upon detection of an event in the tagged sector, the information message will reach the AP promptly. The AP keeps a record of the reporting nodes per sector and directs its charging beam towards the sector that satisfies certain criteria.

In general, there are two mechanisms that influence the performance of the system. The first source of inefficiency is the prompt detection of the event itself and the successful transmission of the information message. Sensor nodes have a limited sensing area, and if the network is sparse, the monitored area is not sufficiently covered. In this case, and assuming that the event does not spread spatially over time, there is a substantial probability of an unnoticed event. Moreover, even if the event is detected promptly, there is no guarantee that the information message will reach the AP. This is due to possible blockage or bad channel conditions. Maximizing the covered area of a sector by sufficiently charged nodes and increasing the probability successful information transmission demands persistent beamforming towards sectors of high numbers of reporting nodes.

The second source of inefficiency is due to the fact that a node transmits an information message only if it is located in the serving sector. Therefore, if the AP spends its time to sectors other than the one that the event takes place, the delivery of information message suffers a considerable delay. In this case, fast (and fair) hopping between sectors is mandatory to reduce the information transmission delay. Note that the two inefficiency mechanisms demand different treatment by the AP. In the following, we join the two contradicting objectives into a reward function of the form

(19) $$J=C_{S}+wC_{D}$$

where $C_{S}$ denotes the reward of beamforming towards sectors of high numbers of reporting nodes, and $C_{D}$ denotes the reward of not delaying energy beamforming of sectors. The factor *w* balances the relative weight of the two objectives. For $C_{S}$, we consider the average number of sufficiently charged sensor nodes per time slot (or sector selection), i.e., $C_{S}=E\left[S\right]$, whereas two alternative reward functions are studied for $C_{D}$. Since the number of nodes that are capable of successfully conveying the information message to the AP is unknown, we replace it with the number of reporting nodes. It is clear that in order for a node to reach the reporting threshold, it must have encountered “good” channel conditions. Thus, reporting is a good indicator of the probability of successful information transmission.

Let us consider a policy of sector selection resulting (up to the time slot *n*) in the sequence of reports

(20) $$R\left(n\right)=[R_{j_{1}}\left(n\right)\;R_{j_{2}}\left(n\right)\;\cdots \;R_{j_{n}}\left(n\right)]$$

where the index $j_{k}\in \left\{1,\ldots ,S\right\}$ indicates the sector served at time slot *k*. Denote by $\nu_{s}\left(n\right)$ the number of times that sector *s* has been selected for service, that is

(21) $$\nu_{s}\left(n\right)=\sum_{k=1}^{n}I\left(j_{k}=s\right)$$

with I(·) the indicator function. Using a time average instead of expectation, we seek a sequential algorithm to maximize

(22) $$\begin{array}{ccc} \bar{R}\left(n\right) & = & \sum_{k=1}^{n}R_{j_{k}}\left(n\right)=\frac{1}{n}\sum_{s=1}^{S}\nu_{s}\left(n\right)\frac{\sum_{\ell =1}^{n}I\left(j_{\ell }=s\right)R_{j_{\ell }}\left(k\right)}{\nu_{s}\left(n\right)}\\ & = & \sum_{s=1}^{S}\frac{\nu_{s}\left(n\right)}{n}{\bar{R}}_{s}\left(n\right)=\sum_{s=1}^{S}f_{s}\left(n\right){\bar{R}}_{s}\left(n\right) \end{array}$$

where $f_{s}\left(n\right)$ is the frequency of serving sector *S* in the first *n* time slots and ${\bar{R}}_{s}\left(n\right)$ is the average number of reporting nodes in sector *s* over the $\nu_{s}\left(n\right)$ selections of the sector. The next sector to be served at time slot n+1 is the one that maximizes $\bar{R}\left(n+1\right)$, that is

(23) $$j_{n+1}^{*}=\arg\max_{j_{n+1}}\bar{R}\left(n+1\right)=\arg\max_{j_{n+1}}\sum_{s=1}^{S}f_{s}\left(n+1\right){\bar{R}}_{s}\left(n+1\right)$$

To this end, we adopt a greedy algorithm that selects the next sector to be served as the one with the highest average of the product $f_{s}\left(\cdot \right){\bar{R}}_{s}\left(\cdot \right)$. Thus, for each s∈{1,…,S}, we form the index

(24) $$A_{s}\left(n+1\right)=\frac{\sum_{k=1}^{n+1}I\left(j_{k}=s\right)f_{s}\left(k\right){\bar{R}}_{s}\left(k\right)}{\nu_{s}\left(n+1\right)}$$

and we serve the sector with the highest value of $A_{s}\left(n+1\right)$. The index $A_{s}\left(n+1\right)$ is computed recursively as

(25) $$A_{s}\left(n+1\right)=\frac{A_{s}\left(n\right)\nu_{s}\left(n\right)+\left({\bar{R}}_{s}\left(n\right)\nu_{s}\left(n\right)+R_{s}\left(n+1\right)\right)/\left(n+1\right)}{\nu_{s}\left(n\right)+1}$$

Note that the number of reporting nodes $R_{s}\left(n+1\right),s=1,\ldots ,S$ is not known, since the sectors have not been served yet. To circumvent this problem, we replace $R_{s}\left(n+1\right)$ with the most recent value, say $R_{s}^{c}\left(n\right)$, of known reporting nodes at time slot *n*. This choice is justified by the small differences in the numbers of reporting nodes for slowly varying channels.

We present now two alternative algorithms that take into account the delay reward $C_{D}$.

### 4.1. Algorithm 1

As was already stated, sectors with small reporting numbers should not be neglected at all. There is always a probability for an event to occur close to a sensor node with “good” channel conditions located in one of these “bad” sectors. Therefore, all sectors should be served, although with different frequency. Our first attempt to maximize the delay reward $C_{D}$ is to use the indices

(26) $$B_{s}\left(n+1\right)=-\frac{1}{d_{s}\left(n+1\right)}$$

where $d_{s}\left(n+1\right)$ denotes the time elapsed since the last time sector *s* was selected for service. Sectors that have not been selected for a long period (large $d_{s}\left(n+1\right)$) exhibit large values of the index $B_{s}\left(n+1\right)$, thus forcing the AP to select them for service as soon as possible. Combining Equations (25) and (26), we propose the algorithm

(27) $$j_{n+1}^{*}=\arg\max_{s}\left\{A_{s}\left(n+1\right)+wB_{s}\left(n+1\right)\right\}$$

A large value of the regulating parameter *w* will force the AP to select sectors in a round robin fashion, whereas a small value of the parameter *w* will force the AP to select sectors with the highest number of reporting nodes. Before summarizing the algorithm, we perform a final adjustment by replacing $R_{s}\left(n+1\right)$ in Equation (25) with $R_{s}^{c}\left(n\right)/max_{k}R_{k}^{c}\left(n\right)$ in order to avoid the bias caused by large numbers of reporting nodes.

Algorithm 1 is described below, with the following defined vectors stored in AP.

- $R^{c}=\left[R_{1}^{c}\left(n\right)R_{2}^{c}\left(n\right)\ldots R_{S}^{c}\left(n\right)\right]$, a vector holding the most recent number of reports per sector.
- $\bar{R}=\left[{\bar{R}}_{1}\left(n\right){\bar{R}}_{2}\left(n\right)\ldots {\bar{R}}_{S}\left(n\right)\right]$, a vector holding the average number of reports per sector.
- $\nu =[\nu_{1}\left(n\right)\nu_{2}\left(n\right)\ldots \nu_{S}\left(n\right)]$, a vector holding the number of selections of each sector.
- $A=[A_{1}\left(n\right)A_{2}\left(n\right)\ldots A_{S}\left(n\right)]$, a vector holding the indices up to the time *n*.
- $D=[d_{1}\left(n\right)d_{2}\left(n\right)\ldots d_{S}\left(n\right)]$, a vector holding the time elapsed since the last service of each sector.

**Algorithm 1:** Dynamic Sector Energy Beamforming

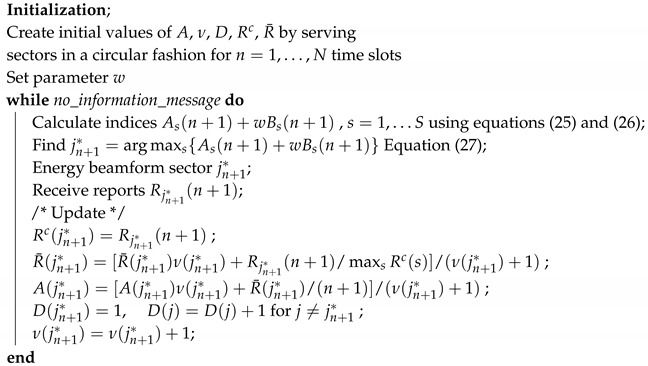



### 4.2. Algorithm 2

The second algorithm borrows ideas from the solution of the multiarmed bandit (MAB) [27] problem and specifically the upper confidence bound (UCB1) algorithm [28]. The MAB problem demonstrates the exploitation versus exploration dilemma and in many aspects fits the sector selection for energy-beamforming problem. In our case, exploitation reflects beamforming towards sectors of high numbers of reports, whereas exploration refers to beamforming towards sectors of bad channel conditions or heavy blockage.

In UCB algorithms, the goal is the maximization of the cumulative reward $\sum_{t=1}^{T}R_{s}\left(t\right)$ over the set of actions *S*, where the random variable $R_{s}\left(t\right)$ has true mean $Q_{s}$ and sample mean ${\bar{Q}}_{s}\left(t\right)=1/t\sum_{\tau =1}^{t}R_{s}\left(\tau \right)$. The algorithm at time step *t* selects the action that maximizes the upper confidence bound

(28) $$s_{t}^{*}=\arg\max_{s\in S}{\bar{Q}}_{s}\left(t\right)+U_{s}\left(t\right)$$

where $U_{s}\left(t\right)$ is a carefully chosen upper confidence bound, so that the true value $Q_{s}$ is less than ${\bar{Q}}_{s}\left(t\right)+U_{s}\left(t\right)$ with high probability. The UCB1 algorithm is based on a heuristic that makes use of Hoeffding’s inequality [29] and sets the upper confidence bound to

(29) $$U_{s}\left(t\right)=\sqrt{\frac{2\log t}{\nu_{s}\left(t\right)}}$$

where $\nu_{s}\left(t\right)$ denotes the number of times action *s* was tried up to the time step *t*. Small values of $\nu_{s}\left(t\right)$ increase the upper bound $U_{s}\left(t\right)$ and consequently force the exploration of action *s*.

The second algorithm we propose uses the same principles as the UCB1 algorithm with a major modification. Since it is desirable to occasionally select sectors with low numbers of reporting nodes, we should not allow the upper bound $U_{s}\left(t\right)$ to vanish as *t* goes to infinity. We periodically “revive” the upper bound so that “bad” sectors have an opportunity to be energy-beamformed. To this end, we define

(30) $$B_{s}\left(n+1\right)=\sqrt{\frac{2\log[(n+1)/f]}{\nu_{s}\left(n+1\right)/f}},\;f=\left\lceil \frac{n+1}{T_{p}}\right\rceil$$

where $T_{p}$ is the time period that adjusts the upper bound and ⌈·⌉ denotes the ceiling operator. The parameter $T_{p}$ has the opposite effect of the parameter *w*. For a large value of $T_{p}$, bad sectors are neglected in the long run, whereas for a small value of $T_{p}$, all sectors are selected sequentially.

Algorithm 2 is summarized as

(31) $$j_{n+1}^{*}=\arg\max_{s}\left\{A_{s}\left(n+1\right)+B_{s}\left(n+1\right)\right\}$$

with $B_{s}\left(n+1\right)$ given by Equation (30) and all other parameters as in Algorithm 1.
**Algorithm 2:** Dynamic Sector Energy Beamforming (MAB approach)
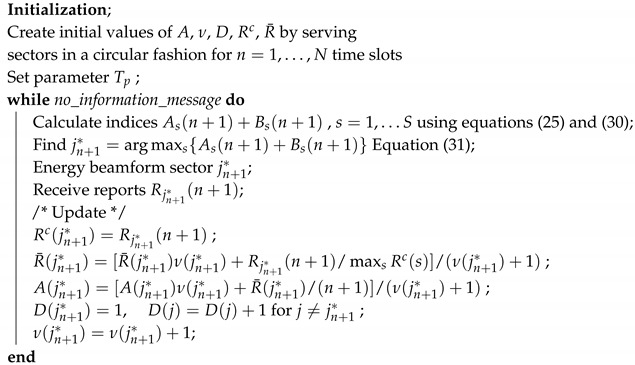


### 4.3. Shifting of Sectors Strategy

**Figure 3:**
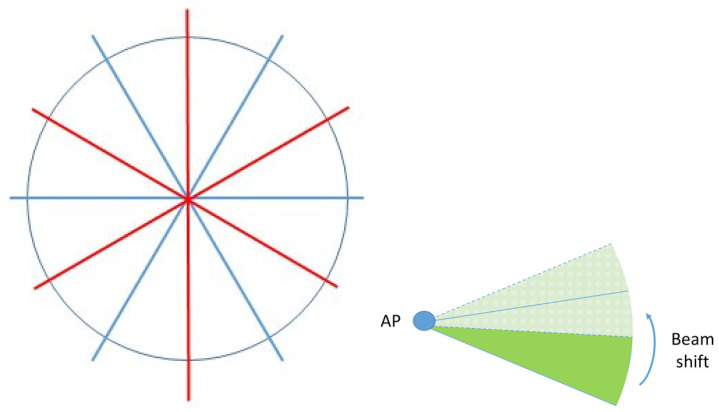
Shifting of sectors strategy.

Due to the presence of the Fejér kernel in the received power formula, energy harvesting is maximum along the main direction of beamforming. Thus, nodes residing at the edges of a sector do not charge at the same level with the rest of the nodes even if they are at the same distance from the AP. A remedy to the problem is to periodically change the direction of the sectors as is shown schematically in Figure 3. For a predefined number of time slots, the AP beamforms according to the “blue” pattern and then switches to the “red” pattern and so on. It is conceivable that this technique can be combined with the aforementioned algorithms for sector selection.

## 5. Numerical Results and Simulations

In this section we assess, the performance of the proposed model and energy-beamforming strategies using simulations. The simulation platform was MATLAB. We used the parameter set of Table 1 taken from [19].

In our simulation model, a base station lies at the center of a cell of radius 10 m, and a linear antenna array of M=8 elements is used to energy beamform 15 sectors. Sensor nodes are randomly deployed forming a Poisson Point Process with intensity λ=1. That is, 314 nodes on average are scattered in the area of interest. Each node is equipped with a battery of N=100 energy levels. Channel gains are modeled as independent random variables following the exponential distribution. However, the channels are slow-varying in the sense that the gains do not change throughout the simulation. All strategies were simulated for 1000 different sensor deployments and channel gains. The simulation horizon is taken equal to 3000 time slots. For each simulation run, an event occurs randomly in the last 1000 time slots. It is further assumed that the event is detected immediately by the nearest node.

**Figure 4:**
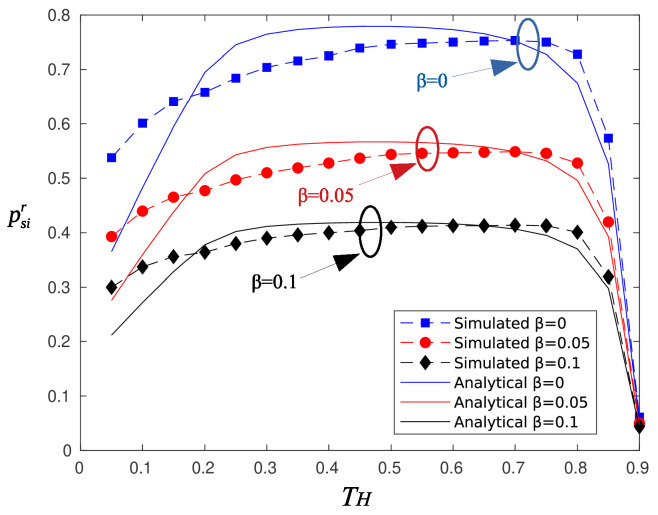
Probability of successful information reception $p_{si}^{r}$ for random selected sectors.

Figure 4 depicts $p_{si}^{r}$ as a function of harvesting period $T_{H}$, for various values of the blocking parameter β. The parameter τ for reporting was set to 90. The analytical expression for $p_{si}^{r}$ is given by Equation (9), and as is observed, there is a good match between the analytical and the simulation results. The $p_{si}^{r}$ curve exhibits a plateau for $T_{H}$ in the range [0.3,0.7]. Smaller values of $T_{H}$ mean less charging time, and therefore, the nodes do not transmit with sufficient power. Large values of $T_{H}$ (greater than 0.8) remedy this problem, but the time period left to transmit the information message reduces considerably. As a result, the information rate increases and the probability of successfully delivering the information message drops.

**Figure 5:**
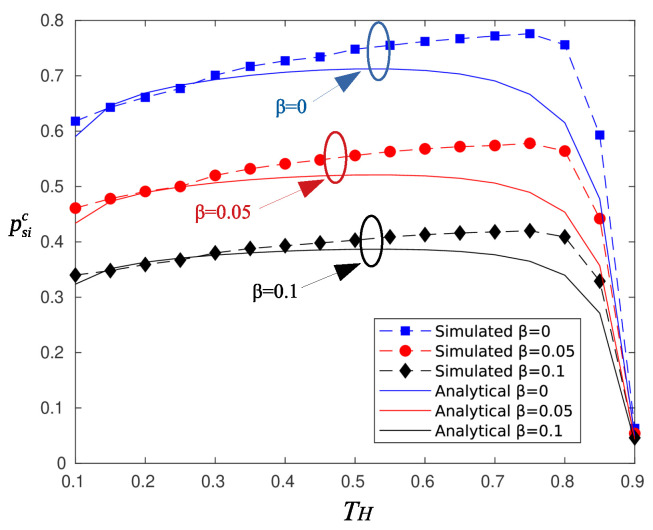
Probability of successful information reception $p_{si}^{c}$ for circular selection of sectors.

Figure 5 depicts $p_{si}^{c}$ for the same parameter set of Figure 4. There is a deviation of the analytical and simulation results for high values of $T_{H}$, and as is observed, there is a slight increase in the probability of successful information reception compared to that of randomly selected sectors.

**Figure 6:**
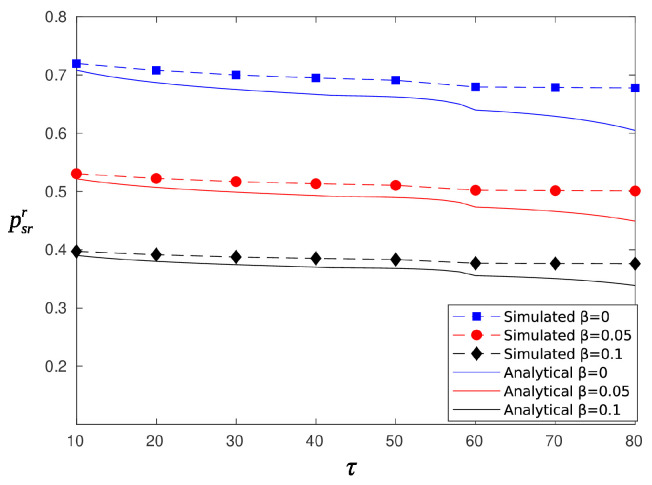
Probability of successful reporting, $p_{sr}^{r}$, for randomly selected sectors.

**Figure 7:**
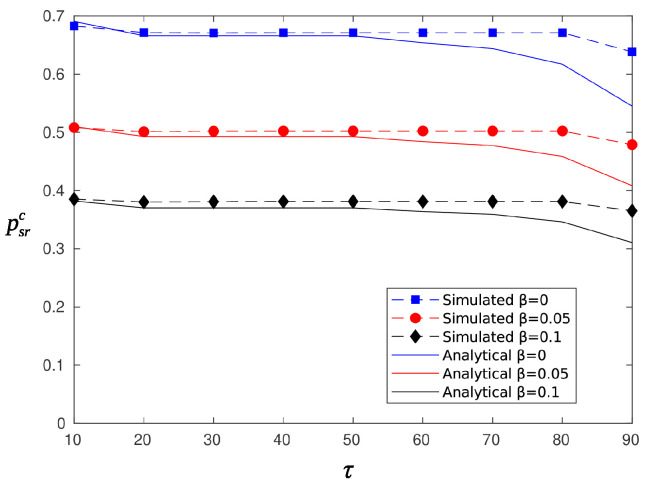
Probability of successful reporting, $p_{sr}^{c}$, for circular selection of sectors.

Figure 6 and Figure 7 depict the probability of a successful report as a function of the threshold τ, for random and circular beamforming, respectively. The parameter $T_{H}$ was set equal to 0.6. Note that Equations (14) and (17) expresses the conditional probability of successful report given that the node is sufficiently charged to attempt reporting, whereas Equations (13) and (16) is the probability of reporting for the random (circular) selection of sectors. Thus, the analytical curves in Figure 6 were obtained using $P\left\{\mathrm{success}\;\mathrm{report},\;\mathrm{rando}\right\}=p_{sr}^{r}p_{r}^{r}$ and those in Figure 7 were obtained using $P\left\{\mathrm{success}\;\mathrm{report},\;\mathrm{circula}\right\}=p_{sr}^{c}p_{r}^{c}$. The simulation and analytical results are in good agreement (error less than 3%) for values of τ less than 60. After τ=60, which is also the percentage of time a node is charging ($T_{H}$), the analytical curves start to deviate from the simulated ones. Comparing random and circular energy beamforming, it is clear that there are no major differences regarding the probabilities of successful information and reporting reception. However, as is shown by the simple delay analysis expressed by Equations (11) and (12), circular energy beamforming is superior regarding the average delay of message delivery. Thus, in the simulations that follow, we consider only the circular case as the benchmark solution.

### 5.1. Epidemic vs. No Epidemic

**Figure 8:**
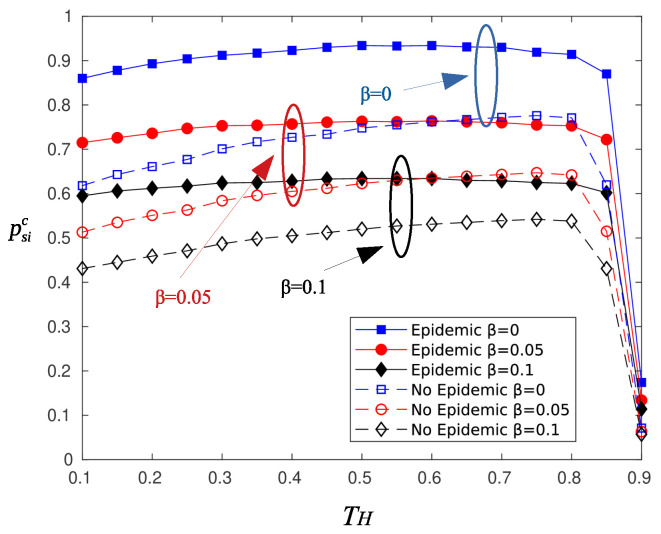
Effect of epidemic mechanism on $p_{si}^{c}$.

**Figure 9:**
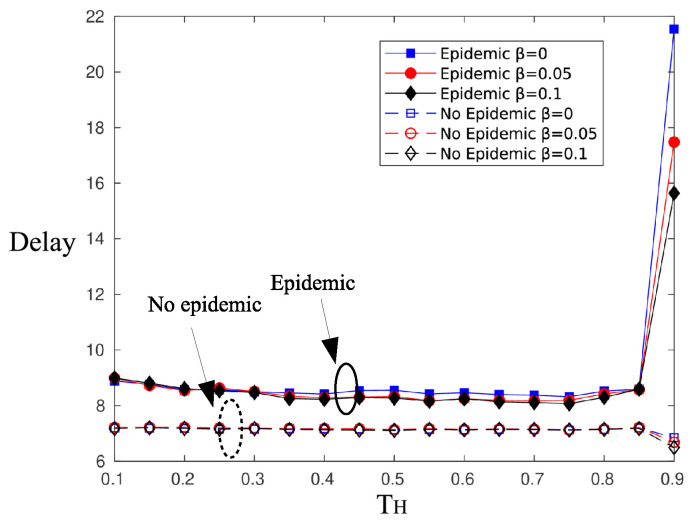
Effect of epidemic mechanism on delay.

Figure 8 and Figure 9 illustrate the effect of the epidemic dissemination of information on the probability and the delay of a successful message reception. As is observed, the probability of successful information reception increases by almost 30%. Neighboring nodes that receive the information transmission act as relays and retransmit the message at first chance. However, receiving the message from a relay node means an additional delay on the message delivery, which accounts for two time slots on average, as is shown in Figure 9.

### 5.2. Alternative Energy-Beamforming Strategies

In the rest of this section, we compare the circular energy-beamforming scheme with alternative strategies proposed in Section 3. In all cases, we did not consider relaying of the information messages.

**Figure 10:**
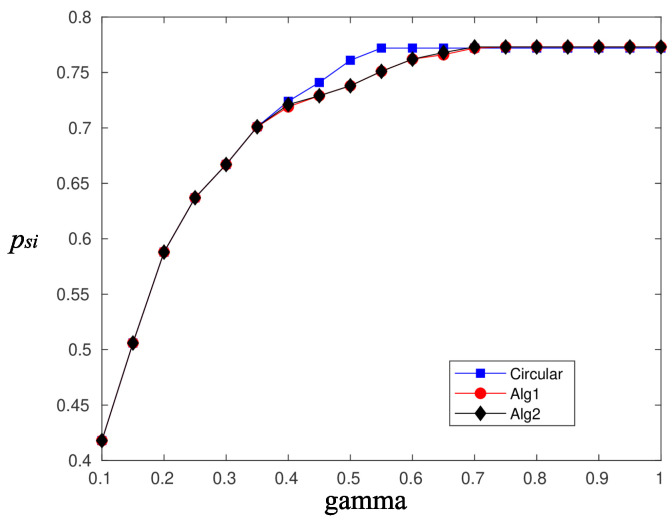
$p_{si}$—Compare sector selection strategies for β=0.

**Figure 11:**
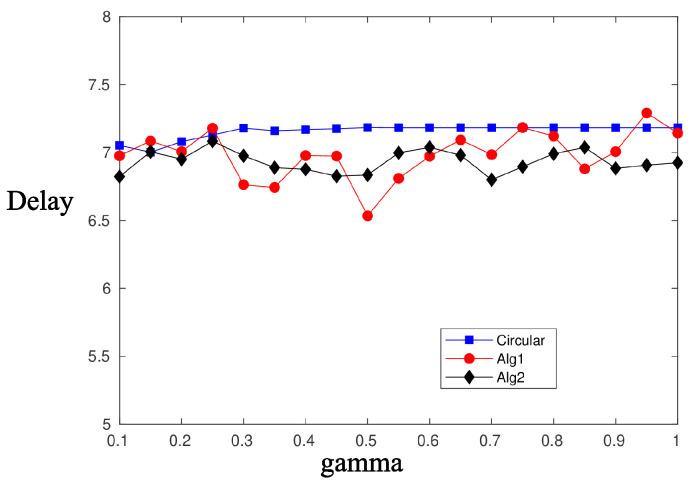
Delay—Compare sector selection strategies for β=0.

**Figure 12:**
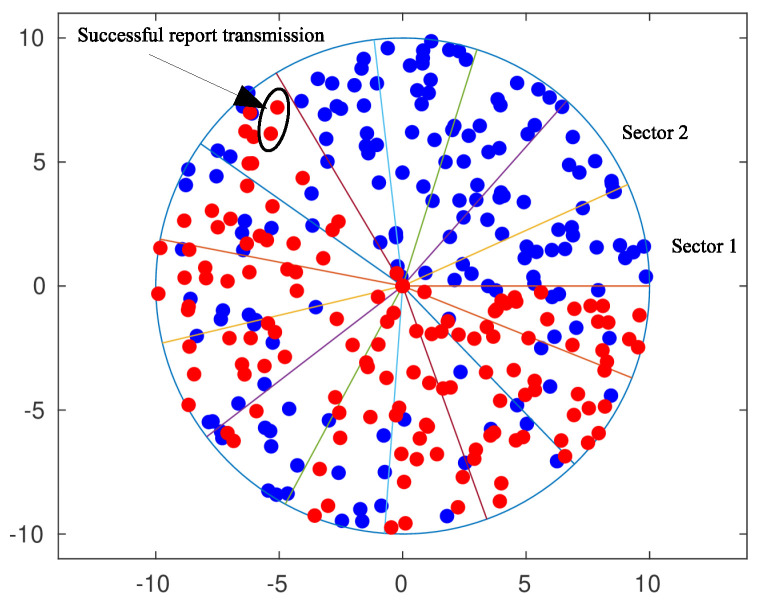
Sensors deployment (red denotes sensors with successful report transmissions).

Figure 10 and Figure 11 depict the performance, in terms of the probability of successful information reception $p_{si}$, and the delay encountered for three strategies (the circular selection of sectors, Algorithms 1 and 2) and for various values of the parameter γ, that is, the charging efficiency of the sensor nodes. All nodes are in LoS with the AP, that is, β=0, and the time duration for energy harvesting is $T_{H}=0.7$. The parameter τ was set equal to 80 and $T_{R}=0.05$. The simulations were obtained using 1000 runs, time horizon 5000 time slots, and an event taking place randomly in the last 15 time slots. It is observed, there are no differences regarding the probability of successful information reception, whereas an advantage seems to be revealed regarding the delay of the information delivery for the non-even selection of sectors. This advantage is expected to be magnified if some sectors are under heavy blockage. To this end, we have simulated two scenarios where, in the first scenario, consecutive sectors 1–5 are subject to blockage and, in the second scenario, equidistant sectors 1, 4, 7, 10, and 13 are subject to blockage. Figure 12 is an illustration of the first scenario, with red color nodes indicating successfully reporting nodes and β=5 for sectors under blockage.

**Figure 13:**
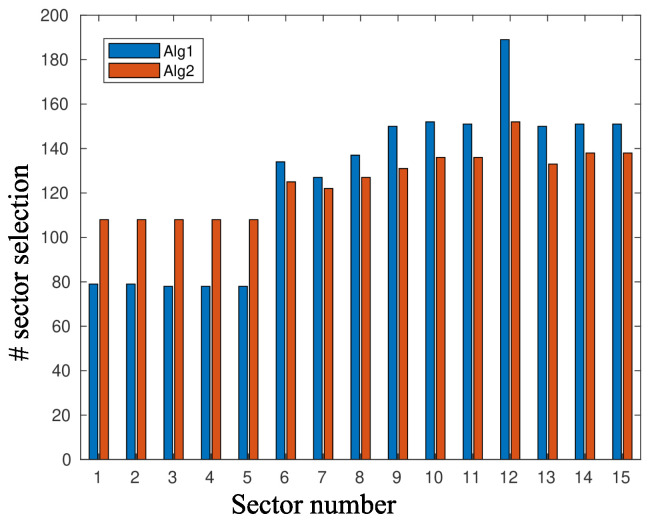
Sector selection distribution, where sectors 1–5 are subject to blockage with β=5.

**Figure 14:**
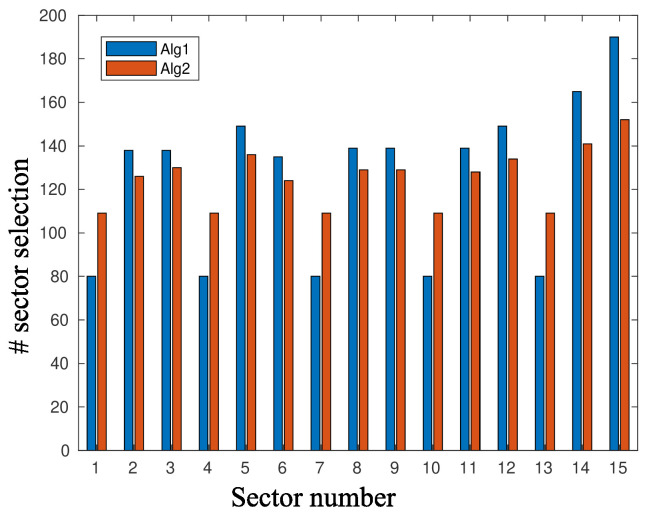
Sector selection distribution, where sectors 1, 4, 7, 10, and 13 are subject to blockage with β=5.

Figure 13 and Figure 14 depict the frequency of sector selection by Algorithms 1 and 2 and for the two scenarios presented above and β=5 for the sectors under blockage. For the first algorithm, the parameter *w* was set equal to 1, whereas $T_{p}=1700$ for the second algorithm. For this choice of the parameter set, Algorithm 1 is more drastic in suppressing the selection of sectors that are subject to blockage.

**Figure 15:**
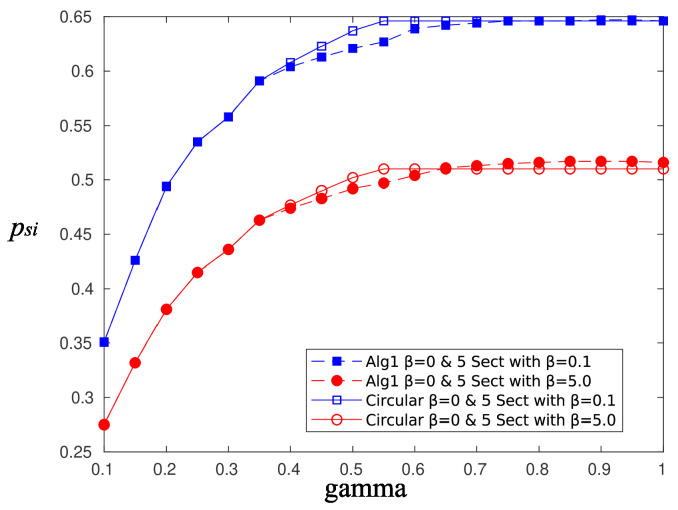
Probability of successful information reception, $p_{si}$, Algorithm 1 vs. circular selection of sectors.

**Figure 16:**
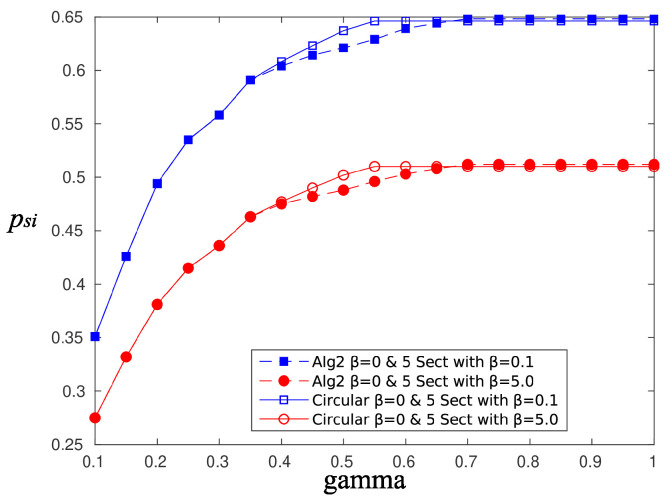
Probability of successful information reception, $p_{si}$, Algorithm 2 vs. circular selection of sectors.

Figure 15 and Figure 16 compare the performance of Algorithm 1 and the circular selection of sectors scheme, in terms of the achievable $p_{si}$ and delay, respectively. The scenario is that of equidistant sectors subject to blockage, and two values of the blockage parameter have been used, β=0.1 and β=5. As is observed, although both algorithms exhibit the same performance in terms of probability of successful information reception, Algorithm 1 is superior regarding the achievable delay. The gain is larger for greater values of β.

**Figure 17:**
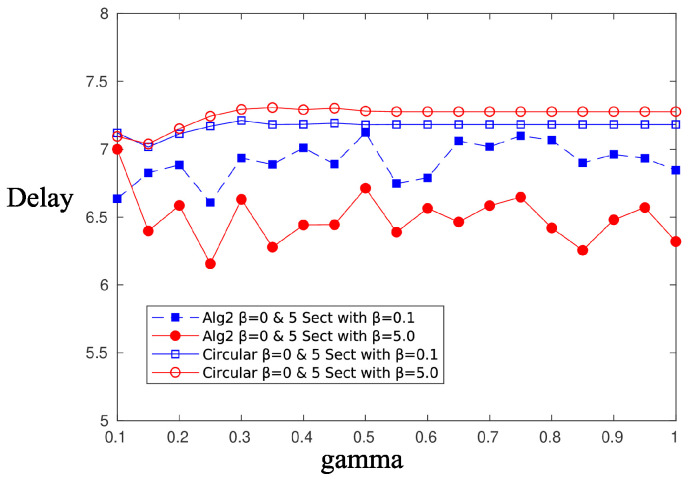
Delay of information reception, Algorithm 2 vs. circular selection of sectors.

A similar performance is obtained using Algorithm 2. Figure 16 and Figure 17 compare the performance of Algorithm 2 and the circular selection of sector scheme, in terms of the achievable $p_{si}$ and delay, respectively. The simulation scenario is the same as in the previous case. Again, Algorithm 2 outperforms the circular sector selection scheme in terms of the achievable delay. In both proposed algorithms, blocked sectors are selected less often, and therefore, the residence time to these sectors is small, thus reducing the overall average delay. If an event occurs within a bad sector, then it is very likely to find depleted sensor nodes, and thus, a successful transmission of the information message is impossible anyway. In the rare case that the event occurs near a node that is capable of transmitting the message successfully, the delay will be large, but its contribution to the average delay is small. Therefore, it is preferable to insist on beamforming “good” sectors, whose sensors have a good chance to transmit successfully, and occasionally give the opportunity to nodes in “bad” sectors to charge and transmit.

**Figure 18:**
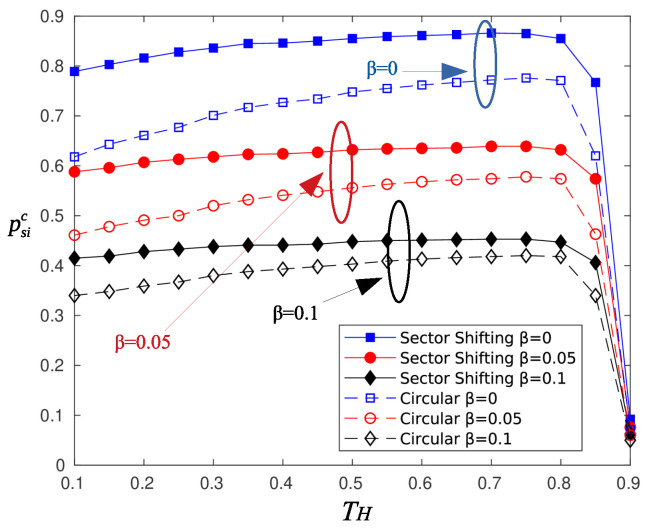
Probability of successful information reception using the sector shifting scheme.

**Figure 19:**
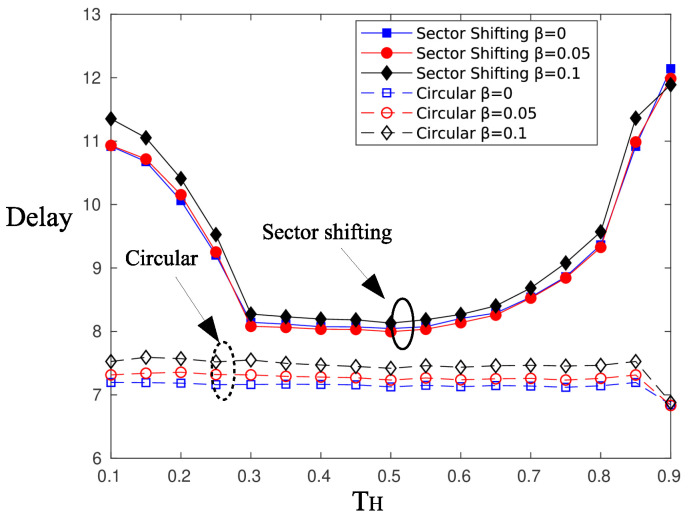
Delay of information reception using the sector shifting scheme.

Next, we assess the performance of the shifting of sectors strategy proposed in Section 4.3. Figure 18 and Figure 19 depict the achievable probability of successful information reception and the average delay, respectively. The simulation parameters are the same as those used to test the epidemic mechanism. The pattern of circular selected sectors is shifted every 15 time slots.

The shifting of sectors strategy outperforms the circular selection in terms of $p_{si}$. Nodes located at the edges of a sector benefit from the shift of the beam pattern, but nodes located at the main direction of the beam become beam edge nodes. Alternating the beam pattern has the overall effect of making battery charging levels independent of the angular position of nodes. It turns out that more nodes are capable of successfully transmitting the information message to the AP, thus increasing $p_{si}$. However, shifting sectors comes with a penalty on latency, and as is observed, there is an increase of 1–4 time slots in the delay of information message delivery.

## 6. Conclusions

A simple sensor reporting protocol for a WPCN network has been proposed. Theoretical expressions for the probability of successful information and successful reporting were provided and verified via model simulations. Sector selection strategies were proposed, based on sensor reporting information, and their performance was assessed using simulations. Having the probability of successful information message reception as a figure of merit, the random selection scheme and the circular selection of sectors exhibit comparable performance to the proposed sector selection strategies. The performance, in terms of the delay of message delivery, is environment-dependent. For environments characterized by diverse blockage, the proposed algorithms outperform the circular (and consequently the random) selection of sectors. Future work will focus on exploiting the proposed reporting mechanism for other crucial tasks than simply declaring the presence of charged nodes. For example, early event indications may be notified to the AP using the proposed reporting mechanism. In this case, the AP may temporarily abandon beamforming all sectors and steer its beam towards the area of interest.

## Figures and Tables

**Table 1:** Parameters used in simulations.

Parameter	Value
$\zeta_{1}$, $\zeta_{2}$, γ, *N*	1500, 0.0022, 1, 100
ρ, θ, α, *M*	10 m, π/15, 2, 8
$P_{0}$, $\sigma^{2}$, λ	−10 db, −30 db, 1
$D_{I}$, $D_{R}$, $W^{\prime }$	512 bits, 128 bits, 1 kHz

## Appendix A

The reporting nodes form a point process, say Φ_2_, which is the original PPP thinned by the probability $p_{r}$. For this thinned process, the spatial distribution of the reporting nodes is given by

(A1) $$f_{R}\left(r,\phi \right)=\frac{\lambda_{\Phi_{2}}\left(r,\phi \right)}{\mu_{\Phi_{2}}}=\frac{\lambda_{\Phi_{2}}\left(r,\phi \right)}{\int_{-\theta }^{\theta }\int_{0}^{\rho }\lambda_{\Phi_{2}}\left(r,\phi \right)rdrd\phi }$$

where $\lambda_{\Phi_{2}}\left(r,\phi \right)$ is the intensity function

(A2) $$\lambda_{\Phi_{2}}\left(r,\phi \right)=\lambda \sum_{i=0}^{\tau }\psi_{i}P\left\{E\left(r,\phi \right)>\left(\tau +1-i\right)E_{s}\right\}$$

and $\mu_{\Phi_{2}}$ the intensity measure of the process. The harvested energy E(r,ϕ) over the period $T_{H}$, for a sensor located at (r,ϕ) is given by

$$P_{k}^{RF}=-\frac{1}{\zeta_{1}}\ln\left(\frac{T_{H}L-E\left(r,\phi \right)}{T_{H}L+E\left(r,\phi \right)\exp\left(\zeta_{1}\zeta_{2}\right)}\right)$$

with

$$P_{k}^{RF}=\frac{P_{0}{\left|g_{k}\right|}^{2}F_{M}\left(\phi \right)}{1+r^{a}}$$

Hence, $E\left(r,\phi \right)>\left(\tau +1-i\right)E_{s}$ provided that

$$P_{k}^{RF}>-\frac{1}{\zeta_{1}}\ln\left(\frac{T_{H}L-\left(\tau +1-i\right)E_{s}}{T_{H}L+\left(\tau +1-i\right)E_{s}\exp\left(\zeta_{1}\zeta_{2}\right)}\right)=\eta_{\tau +1-i}$$

or equivalently

$$|g_{k}{|}^{2}>\frac{\eta_{\tau +1-i}\left(1+r^{a}\right)}{P_{0}F_{M}\left(\phi \right)}$$

Since $|g_{k}{|}^{2}$ is exponentially distributed, and incorporating the LOS probability, we have

(A3) $$\begin{array}{ccc} P\{E\left(r,\phi \right)>\left(\tau +1-i\right)E_{s}\} & = & P\{\left\{|g_{k}{|}^{2}>\frac{\eta_{\tau +1-i}\left(1+r^{a}\right)}{P_{0}F_{M}\left(\phi \right)}\right\}\}\exp\left(-\beta r\right)\\ & = & \exp\left((-\frac{\eta_{\tau +1-i}\left(1+r^{a}\right)}{P_{0}F_{M}\left(\phi \right)}-\beta r\right)) \end{array}$$

Substituting Equation (A3) in Equations (A2) and (A1), we obtain

(A4) $$f_{R}\left(r,\phi \right)=\frac{\lambda_{\Phi_{2}}\left(r,\phi \right)}{\mu_{\Phi_{2}}}=\frac{\lambda \sum_{i=0}^{\tau }\psi_{i}\exp\left(-\frac{\eta_{\tau +1-i}\left(1+r^{a}\right)}{P_{0}F_{M}\left(\phi \right)}-\beta r\right)}{\lambda \theta \rho^{2}p_{r}^{r}}$$

Note that

(A5) $$\begin{array}{ccc} \mu_{\Phi_{2}} & = & \int_{-\theta }^{\theta }\int_{0}^{\rho }\lambda \sum_{i=0}^{\tau }\psi_{i}\exp\left((-\frac{\eta_{\tau +1-i}\left(1+r^{a}\right)}{P_{0}F_{M}\left(\phi \right)}-\beta r\right))rdrd\phi \\ & = & \lambda \sum_{i=0}^{\tau }\psi_{i}\int_{-\theta }^{\theta }\int_{0}^{\rho }\exp\left((-\frac{\eta_{\tau +1-i}\left(1+r^{a}\right)}{P_{0}F_{M}\left(\phi \right)}-\beta r\right))rdrd\phi \\ & = & \lambda \sum_{i=0}^{\tau }\psi_{i}\theta \rho^{2}P\left\{E>\left(\tau +1-i\right)E_{s}\right\}\\ & = & \lambda \theta \rho^{2}p_{r}^{r} \end{array}$$

We return now to the calculation of the probability of successfully receiving a report message, $p_{sr}^{r}$. The message is received correctly by the AP provided that

(A6) $$R_{R}=\frac{D_{R}}{T_{R}}<W^{\prime }\log_{2}\left(1+\mathrm{SNR}\right)$$

where $D_{R}$ is the message length (bits), $T_{R}$ is the time to transmit the report message, $W^{\prime }$ is the bandwidth allocated to a node, and SNR is the signal to noise ratio at the AP. Clearly, the SNR for a node located at (r,ϕ) is

(A7) $$\mathrm{SNR}=\frac{P_{\ell }{\left|g\right|}^{2}F_{M}\left(\phi \right)}{\left(1+r^{a}\right)\sigma^{2}}$$

with $P_{\ell }=\ell E_{s}/T_{R}$, ℓ=1,…,N−τ. As in the case of calculating $p_{si}^{r}$,

(A8) $$\begin{array}{ccc} P\{\mathrm{SNR}\leq x\;|\;P_{\ell }\} & = & P\{\left\{\frac{P_{\ell }{\left|g\right|}^{2}F_{M}\left(\phi \right)}{\left(1+r^{a}\right)\sigma^{2}}\leq x\;|\;P_{\ell }\}\right\}\\ & = & \{P\left\{{\left|g\right|}^{2}\leq \frac{x\left(1+r^{a}\right)\sigma^{2}}{P_{\ell }F_{M}\left(\phi \right)}\;|\;P_{\ell }\}\right\}\\ & = & 1-E_{\Phi_{2}}[\left[\exp(\left(-\frac{x\left(1+r^{a}\right)\sigma^{2}}{P_{\ell }F_{M}\left(\phi \right)})]\right)\right] \end{array}$$

In the last equation, the average is over the thinned process, for which the nodes are distributed according to $f_{R}\left(r,\phi \right)$. Thus,

(A9) $$\begin{array}{c} P\{\mathrm{SNR}\leq x\;|\;P_{\ell }\}\\ =1-\int_{-\theta }^{\theta }\int_{0}^{\rho }\exp\left(-\frac{x\left(1+r^{a}\right)\sigma^{2}}{P_{\ell }F_{M}\left(\phi \right)}\right)f_{R}\left(r,\phi \right)rdrd\phi \\ =1-\frac{1}{\theta \rho^{2}p_{r}^{r}}\int_{-\theta }^{\theta }\int_{0}^{\rho }\sum_{i=0}^{\tau }\psi_{i}\exp\left(-\frac{1+r^{a}}{F_{M}\left(\phi \right)}\left(\frac{x\sigma^{2}}{P_{\ell }}+\frac{\eta_{\tau +1-i}}{P_{0}}\right)-\beta r\right)rdrd\phi \end{array}$$

The probability of successfully receiving a report message $p_{sr}^{r}$ is simply

$$p_{sr}^{r}=P\left\{\mathrm{SNR}>2^{R_{R}/W^{\prime }}-1\right\}=P\left\{\mathrm{SNR}>2^{D_{R}/T_{R}W^{\prime }}-1\right\}$$

Substituting it in Equation (A9) and unconditioning on $P_{\ell }$, we obtain

(A10) $$p_{sr}^{r}=\sum_{\ell =1}^{N-\tau }P\left\{\mathrm{SNR}>2^{D_{R}/T_{R}W^{\prime }}-1\;|\;P_{\ell }\right\}P\left\{P_{\ell }\right\}$$

The probability of transmitting the report message with power $P_{\ell }$, given that the node’s energy level after harvesting exceeds the threshold τ, equals

(A11) $$p_{\ell }=P\left\{P_{\ell }\right\}=\{\left\{\begin{array}{cc} \frac{F_{E}\left(\left(\tau +\ell +1-i\right)E_{s}\right)-F_{E}\left(\left(\tau +\ell -i\right)E_{s}\right)}{1-F_{E}\left(\tau +1-i\right)E_{s}}\; & \ell <N-\tau \\ \frac{1-F_{E}\left(\left(\tau +\ell +1-i\right)E_{s}\right)}{1-F_{E}\left(\left(\tau +1-i\right)E_{s}\right)} & \ell =N-\tau \end{array}\right.$$
